# Impact of Chronic Fluoxetine Exposure on Oocyte Development and Reproductive Outcomes in a Mouse Model

**DOI:** 10.3390/ijms26104858

**Published:** 2025-05-19

**Authors:** Maria D. Tkachenko, Nina M. Alyoshina, Yulia O. Nikishina, Veronika S. Frolova, Denis A. Nikishin

**Affiliations:** 1 Koltzov Institute of Developmental Biology, Russian Academy of Sciences, Moscow 119334, Russia; tkmadm@yandex.ru (M.D.T.); ninalyoshina@gmail.com (N.M.A.); zubova.y@gmail.com (Y.O.N.); 2 Department of Embryology, Faculty of Biology, Lomonosov Moscow State University, Moscow 119234, Russia; frolova.veronika.2014@post.bio.msu.ru

**Keywords:** fluoxetine, oocyte maturation, cytoplasmic maturity, SSRI, ovary, in vitro maturation, blastocyst

## Abstract

Selective serotonin reuptake inhibitors (SSRIs), like fluoxetine, are increasingly used by women of a reproductive age, raising concerns about their impact on oocyte quality and early embryonic development. This study investigated the effects of chronic fluoxetine exposure on oocyte maturation, ovulation, and embryonic development in a mouse model. Female mice were administered fluoxetine via drinking water, and their reproductive outcomes were compared to those of control mice. Oocyte quantity and quality were assessed following superovulation, including the analysis of spindle morphology, chromatin configuration, and maturation markers. In vitro maturation assays were conducted to evaluate the developmental competence of oocytes exposed to fluoxetine. Finally, the impact of fluoxetine on blastocyst formation, litter size, offspring growth, and ovarian reserve was examined. The results show that fluoxetine treatment reduced the number of ovulated oocytes but did not significantly affect oocyte quality or meiotic spindle formation. Fluoxetine exposure impaired cytoplasmic maturation at the germinal vesicle stage, resulting in a lower proportion of fully mature oocytes and reduced in vitro maturation efficiency. While blastocyst numbers were modestly reduced in fluoxetine-treated mice, litter size and offspring ovarian reserve were unaffected. Unexpectedly, offspring of fluoxetine-treated mothers exhibited increased body weight. These findings suggest that while fluoxetine may impair oocyte developmental competence through disruptions in cytoplasmic maturation, it does not severely compromise overall reproductive outcomes or offspring fertility.

## 1. Introduction

The production of developmentally competent oocytes is central to female fertility and the success of species propagation. Oocyte maturation is an intricate process that encompasses a series of tightly coordinated nuclear and cytoplasmic events, including germinal vesicle breakdown (GVBD), meiotic resumption, spindle formation, chromatin remodeling, and cytoplasmic preparation for fertilization and subsequent embryonic development [1]. Disruptions to these processes can reduce the efficiency of reproductive outcomes by impairing ovulation, fertilization, or early embryogenesis. Understanding the mechanisms underlying oocyte and embryo quality is therefore crucial for advancing both natural and assisted reproductive technologies (ARTs).

Selective serotonin reuptake inhibitors (SSRIs), including fluoxetine, are among the most widely prescribed antidepressants globally [2]. Their use has significantly increased among women of a reproductive age, including those planning pregnancy or undergoing fertility treatments [3]. Fluoxetine acts by increasing serotonin levels at synapses in the central nervous system, a mechanism that alleviates depressive symptoms [4]. However, serotonin signaling is not confined to neural tissues; serotonin receptors and transporters are also expressed in peripheral systems, including the ovary, uterus, and early embryo [5,6]. This raises critical questions regarding the potential off-target effects of SSRIs on female reproductive physiology.

While fluoxetine has been associated with delayed conception, reduced fertilization rates, and pregnancy complications in some clinical studies [7,8], the specific effects of chronic fluoxetine exposure on oogenesis and embryo development are not well understood. Previous research has suggested that SSRIs lead to a significant decrease in serotonin in both blood and ovarian tissue, and may modulate ovarian function by interfering with hormonal signaling or oocyte maturation [9,10,11]. Specifically, rodent models have demonstrated that fluoxetine exposure can disrupt various aspects of ovarian function and oocyte quality. For example, studies have shown that after 14 days of exposure to fluoxetine (20 mg/kg/d), mice exhibited disrupted estrous cycles, a reduced number of antral follicles, an increased number of atretic follicles, and decreased ovulation [11]. Concordantly, significant increases in degenerative follicles and damaged zona pellucida were also reported in female Balb/C mice following fluoxetine treatment [12]. These findings are further supported by studies on rats, which have also found negative effects, such as decreased ovulation and impaired ovarian function following fluoxetine exposure [13,14]. At the same time, the evidence on how fluoxetine influences key oocyte quality markers, such as spindle integrity, cytoplasmic maturation, and developmental competence, remains limited. Moreover, the impact of maternal fluoxetine exposure on offspring development, including their reproductive potential, has yet to be systematically explored.

This study was designed to address these gaps by examining the effects of fluoxetine on critical stages of female reproduction in a mouse model, with a focus on (i) the quantity and quality of ovulated oocytes, (ii) the cytoplasmic and nuclear maturation of GV-stage oocytes, and (iii) the developmental competence of oocytes and embryos following fertilization. By combining molecular, morphological, and functional assessments, we sought to elucidate the mechanisms by which fluoxetine might impair oocyte developmental potential and alter early reproductive outcomes. Additionally, we aimed to evaluate whether maternal fluoxetine administration affects offspring growth and ovarian reserve, with implications for intergenerational fertility.

These findings are expected to contribute to our understanding of the broader implications of SSRI use for female reproductive health, particularly in individuals undergoing ART or planning pregnancies, and may guide clinical decision-making regarding the safety of antidepressant use during this critical period.

## 2. Results

### 2.1. Effects of Fluoxetine on the Quantity and Quality of Ovulated MII Oocytes

Female mice treated with fluoxetine via drinking water continued to exhibit regular estrous cyclicity, as confirmed by characteristic changes in the cellular composition observed in vaginal smears. After 10 days of fluoxetine administration, cumulus–oocyte complexes (COCs) were retrieved from oviductal ampullae following superovulation induction. The COCs were treated with hyaluronidase, denuded, and the collected oocytes were analyzed for their quantity and quality.

**Figure 1:**
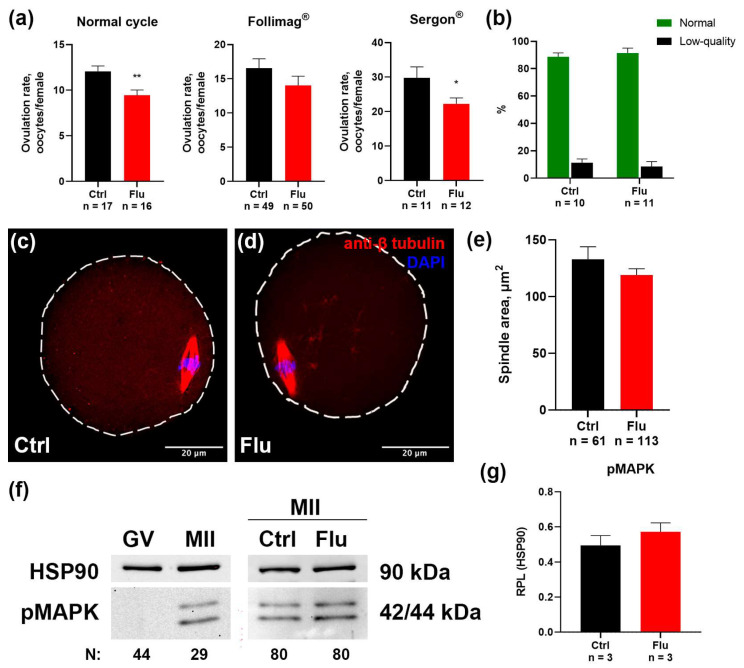
Effects of fluoxetine (Flu) on the quantity and quality of ovulated MII oocytes. (**a**) Number of ovulated MII oocytes obtained from the oviducts of females in a normal cycle and induced by PMSG preparations Follimag^®^ and Sergon^®^. M ± SEM, *—*p* < 0.05, and **—*p* < 0.01, according to the Mann–Whitney U test. (**b**) The ratio of morphologically normal MII oocytes to abnormal low-quality oocytes. (**c**,**d**) MII oocytes from control (**c**) and fluoxetine-treated (**d**) females immunostained with antibodies against β-tubulin to reveal meiotic spindle for morphologic evaluation. (**e**) Values of the lateral projection area of the meiotic spindle of MII oocytes, M ± SEM. (**f**) Western blot analysis of pMAPK protein expression in control GV and MII oocytes, and in MII oocytes from control (Ctrl) and fluoxetine-treated (Flu) females. HSP90 used as internal loading control. (**g**) Quantification of pMAPK relative protein expression level (RPL) calculated in relation to HSP90, M ± SEM.

The mean number of ovulated mature MII oocytes per female is shown in Figure 1a. This negative effect of fluoxetine is consistently observed both when oocytes are obtained in the natural cycle and with the use of different hormonal preparations for superovulation—Follimag^®^ and Sergon^®^ (Figure 1a). In the case of normal cycle, the average number of ovulated oocytes per mouse decreased significantly from 12 in the control to 9.4 in the group treated with fluoxetine, reflecting a 21.6% reduction. In the case of the superovulation protocol using Sergon^®^, the average number of ovulated oocytes per mouse decreased significantly from 29.8 in the control to 22.2 in the group treated with fluoxetine, reflecting a 25.5% reduction.

Morphological analysis of oocyte quality revealed no significant differences in the proportion of normal MII oocytes versus fragmented or degenerating oocytes between the fluoxetine-treated and control groups. In both groups, the proportion of abnormal oocytes remained low and did not exceed 15% (Figure 1b).

To evaluate whether fluoxetine influenced spindle assembly in MII-stage oocytes, oocytes were immunostained with anti-tubulin beta antibodies and analyzed by confocal microscopy (Figure 1c,d). Abnormal spindles were observed only in rare instances in both the control and fluoxetine-treated groups. Furthermore, quantitative measurements of the lateral projection area of the spindles showed no differences between groups (Figure 1e), indicating that fluoxetine did not disrupt spindle organization or the process of spindle assembly.

The possible impact of fluoxetine on nuclear meiotic maturation was assessed by quantifying phosphorylated MAPK, a marker of meiotic progression. This active kinase is present in MII oocytes but absent in GV oocytes prior to GVBD (Figure 1f). Levels of phosphorylated MAPK in MII oocytes collected from fluoxetine-treated females were similar to those in control oocytes (Figure 1g).

Taken together, these findings suggest that the observed reduction in the number of ovulations in fluoxetine-treated animals is not associated with apparent disruptions in oocyte maturation processes, as evidenced by normal spindle formation, chromatin organization, and MAPK activation in MII-stage oocytes.

### 2.2. Effects of Fluoxetine on GV Oocyte Maturation and Cumulus Expansion In Vitro (IVM)

**Figure 2:**
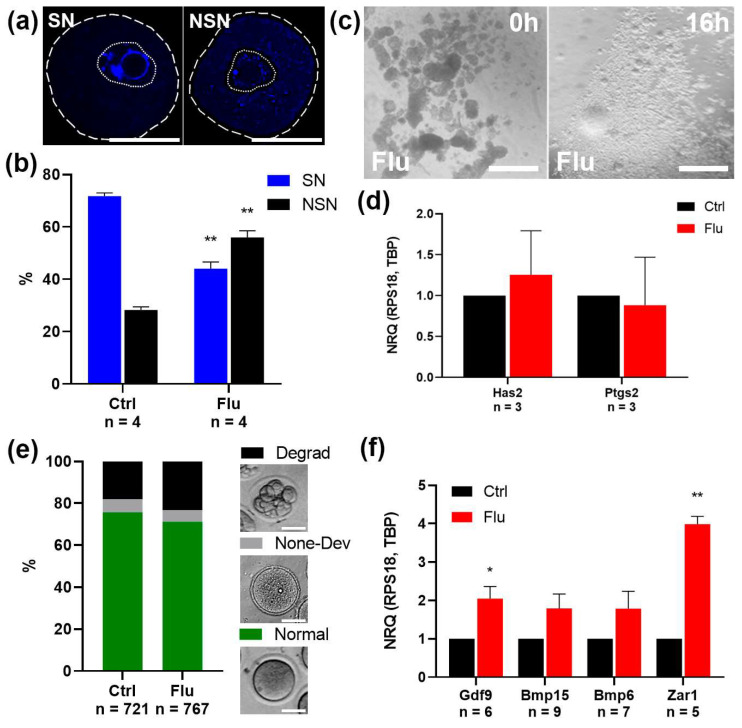
Effects of fluoxetine on GV oocyte maturation and cumulus expansion in vitro. (**a**) Visualization of SN and NSN chromatin conformation in GV oocytes. The dashed line indicates the contours of the oocyte; the dotted line indicates the boundaries of the germinal vesicle. Scale bar 50 μm. (**b**) Analysis of the ratio of SN and NSN oocytes derived from ovaries of control and fluoxetine-treated females, M ± SEM. **—*p* < 0.01, according to two-way ANOVA analysis. (**c**) Microphotographs of oocyte–cumulus complexes isolated from ovaries of fluoxetine-treated females before (0 h) and after (16 h) cumulus expansion in vitro. Loosening of the cumulus cell layer around the oocytes is visible. Scale bar 500 μm. (**d**) qPCR analysis of mRNA expression of *Has2* and *Ptgs2*, functional markers of cumulus cells. NRQ was calculated relative to the expression of reference genes *Rps18* and *Tbp*, and normalized to the control sample taken as 1. M ± SEM. (**e**) The ratio of morphologically normal MII oocytes, non-developing GV oocytes, and degenerated oocytes obtained in an IVM experiment. Scale bar 50 μm. (**f**) qPCR analysis of mRNA expression of oocyte-derived growth factors *Gdf9*, *Bmp6*, and *Bmp15*, and negative marker of cytoplasmic maturity, *Zar1*. NRQ was calculated relative to the expression of reference genes *Rps18* and *Tbp*, and normalized to the control sample taken as 1. M ± SEM, *—*p* < 0.05, and **—*p* < 0.01 according to the one-sample *t* test.

To assess the potential effects of fluoxetine on earlier stages of oogenesis, we analyzed the maturation of GV-stage oocytes obtained 36 h after superovulation induction. Morphological analysis of chromatin structure revealed a significant reduction in the proportion of fully mature SN (surrounded nucleolus) oocytes in the fluoxetine-treated group (42%) compared to the control group (72%) (Figure 2a,b). This result suggests a delay in the cytoplasmic maturation of GV oocytes, which may underlie the observed decrease in ovulated oocyte numbers in fluoxetine-treated females.

To further evaluate the developmental potential of GV oocytes, we assessed their capacity for in vitro maturation (IVM) as oocyte–cumulus complexes. Both the control and fluoxetine-treated groups demonstrated successful cumulus expansion in response to EGF supplementation, as confirmed by morphological analysis (Figure 2c). Additionally, quantitative PCR analysis of cumulus-specific marker genes, *Has2* and *Ptgs2*, revealed no significant differences in gene expression between the experimental and control groups (Figure 2d). This indicates that fluoxetine does not disrupt granulosa cell differentiation into cumulus cells.

Morphological analysis of oocytes following IVM, however, revealed a decrease in the proportion of mature MII-stage oocytes in the fluoxetine-treated group and an associated increase in the proportion of degenerated oocytes (Figure 2e). This may be attributed to the incomplete cytoplasmic maturation of GV oocytes observed in fluoxetine-treated females.

To investigate the molecular characteristics of fluoxetine-exposed MII-stage oocytes, we analyzed the expression levels of key oocyte growth-factor mRNAs, including *Gdf9*, *Bmp6*, and *Bmp15*, via real-time qPCR (Figure 2f). Interestingly, mRNA levels for these genes were slightly elevated in the fluoxetine-treated group compared to controls. However, the level of *Zar1*, a negative marker of cytoplasmic maturity, was also found to be higher in the fluoxetine-treated group (Figure 2f). This indicates that the fluoxetine-treated MII oocytes displayed a less-mature cytoplasmic state compared to the control group.

In summary, fluoxetine treatment led to impaired cytoplasmic maturation at the GV stage, resulting in compromised oocyte quality at the MII stage.

### 2.3. Effects of Fluoxetine on Reproduction and Developmental Potential

**Figure 3:**
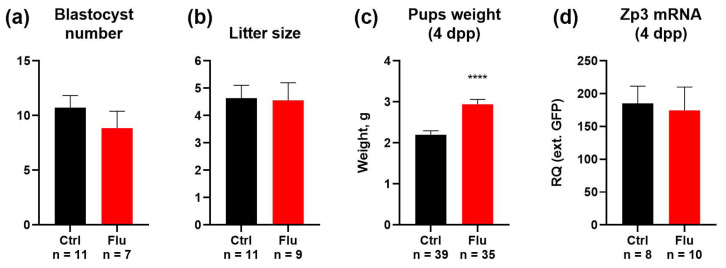
Effects of fluoxetine on reproduction and developmental potential, M ± SEM. (**a**) Number of blastocysts contained in the uterine horns on day 3.5 of development, M ± SEM. (**b**) Litter size of control females and females taking fluoxetine during pregnancy, M ± SEM. (**c**) Weight of mouse pups on the 4th day of life (4 dpp). M ± SEM, ****—*p* < 0.0001, according to the Mann–Whitney U test. (**d**) qPCR analysis of 4 dpp females’ ovarian reserve by expression of the marker *Zp3* normalized to an external RNA standard (*GFP*), M ± SEM.

To evaluate the effects of fluoxetine on embryonic development and offspring outcomes, we monitored the number of blastocysts present in uterine horns following natural pregnancies, confirmed by the presence of vaginal plugs. The average number of blastocysts decreased from 10.7 in the control to 8.8 in the group of females treated with fluoxetine (17.8% reduction) (Figure 3a). This reduction aligns with the observed decrease in the number of ovulated MII oocytes.

Despite this reduction in blastocyst numbers, the litter size in both groups showed no significant differences, indicating that fluoxetine treatment did not impair the ability of embryos to develop to term (Figure 3b). Interestingly, the average body weight of pups born from fluoxetine-treated females was significantly increased by 31.8% compared to pups from the control group (Figure 3c).

To assess whether maternal fluoxetine exposure affected the ovarian reserve of female offspring, 4-day postpartum (4 dpp) female pups were analyzed for the expression of the oocyte-specific marker *Zp3* in ovaries. Quantitative analysis revealed no significant differences in ovarian reserve between female offspring born to fluoxetine-treated mothers and those born to untreated controls (Figure 3d).

These findings suggest that while fluoxetine treatment during pre-conception and pregnancy results in a modest reduction in blastocyst numbers, it does not adversely affect litter size, enhances offspring body weight, and does not critically impair the ovarian reserve of female progeny.

## 3. Discussion

In this study, we investigated the effects of fluoxetine on oocyte maturation, ovarian function, and developmental potential in female mice, providing novel insights into how this widely prescribed antidepressant influences key stages of reproduction. Our findings reveal that fluoxetine treatment does not significantly disrupt oocyte quality or meiotic spindle formation at the MII stage, yet it reduces the number of ovulated oocytes and impairs cytoplasmic maturation during earlier stages of oogenesis. Additionally, fluoxetine exposure was associated with a modest reduction in blastocyst numbers but did not adversely affect overall litter size or offspring ovarian reserve. Surprisingly, we observed an increase in pup body weight, representing a potentially unexpected maternal drug-related effect.

The observed reduction in ovulated MII oocyte numbers following fluoxetine treatment aligns with prior studies linking selective SSRIs to impaired ovarian function [9,15]. This effect manifests to varying degrees both in natural cycles and during hormonal induction of superovulation (Figure 1a). Notably, Follimag exhibited the least pronounced effect in the context of a less effective superovulation. Conversely, Sergon demonstrated a more pronounced and statistically significant effect of fluoxetine against the backdrop of a more potent induction of follicular growth. These differences may stem from the varying compositions of the hormonal preparations, specifically their differing follicle-stimulating activity, which leads to different degrees of follicular growth activation [16]. Despite this quantitative deficit in oocyte number, the quality of the ovulated oocytes remained largely unaffected. This is evidenced by the consistent proportions of normal MII oocytes and intact spindle morphology observed in both the fluoxetine-treated and control groups. The maintenance of spindle structure and chromatin organization suggests that the effects of fluoxetine may primarily target processes upstream of meiotic completion, such as cytoplasmic or nuclear maturation during the GV stage [17].

Our results highlight a potential mechanism by which fluoxetine impairs oocyte developmental potential: through disruptions in cytoplasmic maturation at the GV stage. The lower proportion of SN chromatin configuration in GV oocytes from fluoxetine-treated females indicates delayed or incomplete cytoplasmic maturation [18]. This delay could explain the reduced efficiency of in vitro maturation to the MII stage observed in this study. Moreover, molecular analysis of oocyte-derived growth factor mRNAs (*Gdf9*, *Bmp6*, *Bmp15*) in fluoxetine-treated oocytes revealed slightly elevated levels, which might reflect a compensatory response to developmental insufficiency [19]. Similarly, the increased expression of *Zar1*, a marker of cytoplasmic immaturity [20], further supports the notion that fluoxetine-treated oocytes exhibit a less mature state. Collectively, these findings emphasize the critical role of cytoplasmic maturation in establishing oocyte competence for subsequent embryonic development. We are convinced that these observed effects are likely mediated through the primary mechanism of fluoxetine action: the blockade of SERT activity at the oocyte surface membrane [5]. As previously demonstrated [9], SERT inhibition by fluoxetine leads to a depletion of the cytoplasmic serotonin pool within the oocyte. This reduction in intracellular serotonin may, in turn, disrupt non-receptor-mediated intracellular serotoninergic signaling pathways that are critical for normal oocyte maturation. One potential mechanism through which serotonin depletion could exert its negative influence is via the disruption of protein serotonylation, the covalent modification of proteins by serotonin. This process has been shown to regulate the function of numerous proteins, including histones [21]. Alterations in epigenetic patterns could, therefore, impact gene expression profiles necessary for proper cytoplasmic maturation in oocytes [22]. Furthermore, serotonin is a known modulator of mitochondrial function [23,24]. We hypothesize that fluoxetine-induced serotonin depletion may impair mitochondrial function within the oocyte, thereby contributing to the observed defects in cytoplasmic maturation. Further research is warranted to fully elucidate the specific intracellular targets of serotonin action in oocytes and should focus on detailed mechanistic investigations to dissect the complex interplay between serotonin, its derivatives, and intracellular signaling pathways during oocyte maturation.

The impact of fluoxetine extended beyond oocyte maturation and ovulation, influencing embryonic development and offspring characteristics. Fluoxetine-treated females exhibited a modest 17.5% reduction in blastocyst numbers, consistent with the decreased number of ovulated oocytes. Despite this reduction, litter sizes in fluoxetine-treated and control groups were comparable, indicating that the embryos that successfully implanted retained the capacity to reach term. However, it should be noted that quantitative changes in female productivity at the ovulation stage are not critical, and therefore may not be as pronounced at subsequent stages of embryonic development, such as blastocysts and newborn mice. The observed inconsistency in the changes in the number of blastocysts and litter size in the experiment is likely attributable to an insufficiently large sample size. In previous studies using animal models, it was found that the number of pups weaned decreased, corresponding to increased neonatal mortality in SSRI-treated groups [25]. Moreover, population-based linked health data have shown that prenatal SSRI exposure is associated with an increased risk of low birth weight [26], whereas in our study, the weight of mice at 4 days of age was statistically significantly increased in the fluoxetine group. The observed discrepancies with previously published data may stem from variations in experimental design, including differences in the specific substrains of laboratory mice employed and the time point at which pup weight was assessed. However, evidence exists that supports our findings. Chronic exposure studies involving the long-term administration of SSRIs in mice have described a propensity for obesity and metabolic disturbances, including insulin resistance and glucose intolerance [27]. These complex metabolic alterations can influence the dams’ ability to nurture their offspring [28] and can potentially cause a higher birth weight [29]. Therefore, the observed changes in pup weight may be a consequence of the broader metabolic impact of fluoxetine on maternal metabolic status. Importantly, there was no significant effect on the ovarian reserve of female offspring, as indicated by comparable levels of *Zp3* mRNA expression in neonatal ovaries across treatment groups. This suggests that maternal fluoxetine exposure does not critically impair the reproductive potential of future generations under the conditions studied.

Our findings have several broader implications in the contexts of reproductive biology and clinical practice. First, while fluoxetine-induced delays in cytoplasmic maturation may impair oocyte developmental competence in vitro, the absence of significant defects in spindle stability and chromatin organization suggests that fluoxetine-treated oocytes maintain sufficient structural integrity for fertilization and embryogenesis under physiological conditions. Second, the reduction in blastocyst numbers highlights the need to carefully consider the use of fluoxetine in women undergoing ART, where a reduced oocyte yield can diminish the overall success rate. However, the lack of a pronounced negative impact on litter size and ovarian reserve is reassuring for women taking fluoxetine during pregnancy or preconception, as it suggests no substantial long-term impact on offspring fertility.

A key limitation of this study is the inability to fully delineate the molecular mechanisms driving the observed alterations in oogenesis and embryogenesis. Further research is needed to explore whether fluoxetine’s effects on oocyte maturation result from direct action on ovarian serotonin systems or indirect alterations in endocrine and metabolic pathways. Additionally, the dose-dependent effects of fluoxetine on reproduction remain unexplored and should be prioritized in future studies.

In summary, our findings provide new evidence that fluoxetine treatment impairs oocyte quantity and competency through disruptions in cytoplasmic maturation while preserving structural integrity and developmental potential of fertilized embryos. Although blastocyst production is modestly reduced, the absence of negative effects on litter size, offspring reproductive potential, and maternal health points to a nuanced role of fluoxetine in reproduction. These insights are critical for understanding the reproductive risks associated with SSRI use and evaluating their implications for fertility and ART outcomes.

## 4. Materials and Methods

### 4.1. Experimental Animals and Chemicals

Female ICR mice were obtained from the Laboratory Animal Center of the Koltzov Institute of Developmental Biology RAS and used for all animal experiments. Mice were housed under controlled environmental conditions (22–24 °C, 14 L:10 D photoperiod) with ad libitum access to food and water. All procedures were performed in accordance with the Council of the European Communities Directive of 24 November 1986 (86/609/EEC) and approved by the Commission on Bioethics of the Koltzov Institute of Developmental Biology of the Russian Academy of Sciences.

Fluoxetine hydrochloride (Macklin Inc., Shanghai, China; catalog number F844356) was used in this study. It was dissolved in drinking water at a concentration of 0.13 mg/mL, as previously described [30,31].

### 4.2. Superovulation Protocol and Oocyte and Embryo Retrieval

Mature female mice (2 months old) were superovulated via intraperitoneal injection of 5 IU of PMSG (Follimag, Mosagrogen, Moscow, Russia; or Sergon, Bioveta Inc., Ivanovice na Hané, Czech Republic) between 9:00 PM and 10:00 PM. Forty to forty-six hours post-PMSG injection, mice received an intraperitoneal injection of 5 IU hCG at 7:00–8:00 PM. GV oocyte isolation: Ovaries were harvested 36 h post-PMSG injection, rinsed in ice-cold dPBS, and placed in L-15 medium (Thermo Fisher Scientific Inc., Waltham, MA, USA), pre-warmed to 37 °C. Germinal vesicle (GV)-stage oocytes were released by puncturing antral follicles with a 29 G needle. MII oocyte isolation: 16 h post-hCG injection, oviducts were dissected and placed in ice-cold dPBS. Cumulus–oocyte complexes (COCs) were collected from the ampulla using a V-Denupet Handle (Vitromed GmbH, Jena, Germany). COCs were then transferred to a four-well plate (Thermo Fisher Scientific Inc., Waltham, MA, USA) containing L-15 medium supplemented with 80 U/mL hyaluronidase (Merck KGaA, Darmstadt, Germany) for 15 min to remove cumulus cells. Denuded metaphase II (MII)-stage oocytes were then washed three times for 5 min each in L-15 medium. Blastocyst Collection: For blastocyst collection, female mice were placed with males overnight. Detection of a vaginal plug was considered 0.5 days post-coitum (dpc). Blastocysts were collected at 3.5 dpc by flushing the uterus with dPBS.

In assessing the quality of oocytes, the following criteria were applied. GV oocyte was defined by the presence of a germinal vesicle with a dense nucleolus-like body. Mature oocytes of good quality were defined by the clear homogeneous cytoplasm without expressed inclusions. Poor-quality oocytes were defined by granular cytoplasm and fragmentation (Figure 2e).

### 4.3. In Vitro Oocyte Maturation and Cumulus–Oocyte Complex Expansion

Cumulus–oocyte complexes were isolated by puncturing mouse ovaries 24 h after PMSG injection in L-15 medium preheated to 37 °C. Non-expanded COCs were collected using a V-Denupet Handle and placed in DMEM/F12 medium (PanEco, Moscow, Russia) supplemented with 3 mM L-glutamine (PanEco, Moscow, Russia), 10% FBS (PanEco, Moscow, Russia), gentamicin (PanEco, Moscow, Russia), and 20 ng/mL epidermal growth factor (EGF) (PanEco, Moscow, Russia). Expansion status and oocyte maturation were evaluated by microscopic examination after overnight culture at 37 °C under a humidified atmosphere of 95% air and 5% CO_2_.

### 4.4. Immunohistochemistry

Isolated oocytes were fixed with 4% paraformaldehyde (PFA) in PBS for 1 h at room temperature, permeabilized with PBS containing 0.1% Triton X-100 (Merck KGaA, Darmstadt, Germany) (PBST), and treated with 0.5% SDS for 1 min to remove the zona pellucida. Samples were then washed in PBST, blocked with 5% FBS in PBST, and incubated for 1 h at room temperature with a mouse anti-β-tubulin antibody (1:10,000; ABClonal, Wuhan, China; catalog number AC021). After washing in PBST, samples were stained with goat anti-mouse IgG secondary antibody conjugated with CF 568 (1:500; Merck KGaA, Darmstadt, Germany; catalog number sab4600312). DNA was counterstained with DAPI (Merck KGaA, Darmstadt, Germany) to visualize GV chromatin configuration. Samples were washed three times with PBST and mounted in Mowiol (Merck KGaA, Darmstadt, Germany) for confocal microscopy.

### 4.5. Image Analysis

Samples were analyzed using a Zeiss LSM 880 Airyscan confocal laser-scanning microscope (Carl Zeiss, Jena, Germany) using consistent settings for objectives, pinhole size, laser intensity, and detector sensitivity across all experiments. Images were processed and analyzed using FIJI software (ImageJ 2.9.0/1.54f, open source, available at https://imagej.net/software/fiji/ (URL accessed on 19 March 2025)). Spindle size was measured using the area tool for regions of interest.

### 4.6. Western Blotting

GV and MII oocyte samples (50–100 oocytes per sample) were lysed in RIPA buffer supplemented with a protease/phosphatase inhibitor cocktail (Cell Signaling Technology, Inc., Danvers, MA, USA; catalog number 5872S). Lysates were denatured in Laemmli buffer for 30 min at 37 °C. SDS-PAGE gel electrophoresis and protein transfer were performed as previously described [9]. The following primary antibodies were used: rabbit mAb phospho-p44/42 MAPK (Thr202/Tyr204) (1:1000; Cell Signaling Technology, Inc., Danvers, MA, USA; catalog number 20G11) and rabbit anti-HSP90AB1 (1:5000; Merck KGaA, Darmstadt, Germany; catalog number sab4300541). Horseradish peroxidase-conjugated goat anti-rabbit IgG (1:50,000; Jackson ImmunoResearch Labs, West Grove, PA, USA; catalog number 111-035-144) was used as a secondary antibody. After washing in TBST, protein bands were visualized using enhanced chemiluminescence (0.1 M Tris-HCl, pH 8.5, 12.5 mM luminol, 2 mM coumaric acid, and 0.09% H_2_O_2_). Band intensities were quantified using Image Lab software Version 6.0.1 build 34 (Bio-Rad Laboratories, Inc., Hercules, CA, USA) and normalized to HSP90 protein levels.

### 4.7. Quantitative Polymerase Chain Reaction (qPCR)

Total RNA was extracted using the guanidine isothiocyanate method with an RNA extraction reagent (Evrogen, Moscow, Russia). RNA samples were treated with DNase I (Thermo Fisher Scientific, Waltham, MA, USA). Purified RNA was reverse transcribed using random hexadeoxynucleotides and Magnus transcriptase (for oocytes) or Moloney murine leukemia virus reverse transcriptase (for granulosa cells and ovaries) (Evrogen, Moscow, Russia), according to the manufacturer’s instructions. qPCR was performed using a StepOnePlus Real-Time PCR System (Thermo Fisher Scientific, Waltham, MA, USA) and a qPCRmix-HS SYBR + HighROX kit (Evrogen, Moscow, Russia). Gene expression levels were normalized to the reference genes *Rps18* and *Tbp* using the 2−∆∆Ct method, with consistent threshold conditions for all genes. Primer sequences are listed in Table 1.

### 4.8. Statistical Analysis

Statistical analyses were performed using GraphPad Prism 9.0 software (GraphPad Software, San Diego, CA, USA). A *p*-value of less than 0.05 (*p* < 0.05) was considered statistically significant. Data are presented as mean ± standard error of the mean (SEM) unless otherwise stated.

## Figures and Tables

**Table 1:** Primer sequences used for qPCR.

Gene Name	NCBI Gene ID	Forward Primer	Reverse Primer
*Bmp15*	12155	GAATCTGATGCCTCTTGTCCTT	ATGGCATGGTTGGGTGAAT
*Bmp6*	12161	ACCGTACTTTGTGGCAGAGC	GAAAAGGCAAAGAGCAGAGTTAG
*Gdf9*	14566	GCCTCCCCGACCTTTAGA	TGCCTCAGACTCCACATTTTC
*GFP*	-	CATGGCCGACAAGCAGAAGAAC	GGCGGCGGTCACGAACTC
*Has2*	15117	GCGGAAGAAGGGACAACA	TGCGGTGCCACAATACTG
*Ptgs2*	19225	CCCTCCGGTGTTTGTCCTT	CCTGCAGCATTTTTCATCTTGTA
*Rps18*	20084	AAGAAAATTCGAGCCCATAGAGG	TAACAGCAAAGGCCCAGAGACT
*Tbp*	21374	GTAGCGGTGGCGGGTATCT	CGTCTTCAATGTTCTGGGTTATCT
*Zar1*	317755	GTGATTCGGATGCCCCTCG	GGGCAGGCACATCTAGTTCTT
*Zp3*	22788	TGCCAGACCCGAACTCCT	TAGCTGGCGCGACTTTGA

## Data Availability

The original contributions presented in this study are included in the article. Further inquiries can be directed to the corresponding author.

## Abbreviations

The following abbreviations are used in this manuscript:

ARTs	Assisted reproductive technologies
COC	Cumulus–oocyte complex
GV	Germinal vesicle (meiotic maturation stage)
GVBD	Germinal vesicle breakdown
IVM	In vitro maturation
MII	Metaphase II (meiotic maturation stage)
NSN	Non-surrounded nucleolus (chromatin configuration)
PCR	Polymerase chain reaction
PMSG	Pregnant mare serum gonadotropin
RAS	Russian Academy of Sciences
SDS-PAGE	Sodium dodecyl sulfate–polyacrylamide gel electrophoresis
SN	Surrounded nucleolus (chromatin configuration)
SSRI	Selective serotonin reuptake inhibitor
